# Preface to the BMC-CIBB 2015-16 special issue

**DOI:** 10.1186/s12859-018-2176-4

**Published:** 2018-07-09

**Authors:** Claudia Angelini, Andrea Bracciali, David Gilbert, Riccardo Rizzo

**Affiliations:** 1 Istituto per le Applicazioni del Calcolo - Consiglio Nazionale delle Ricerche, Via Pietro Castellino 111, Napoli, 80131 Italy; 2 Computing Sciences and Mathematics University of Stirling, FK9 4LA, Stirling, UK; 3 Department of Computer Science, St John’s Building, Brunel University London, Uxbridge, Middlesex, London, UB8 3PH UK; 4 Istituto di Calcolo e Reti ad Alte Prestazioni, Consiglio Nazionale delle Ricerche, Via Ugo La Malfa, 153, Palermo, 90146 Italy

This BMC special issue collects a selection of eleven revised and extended papers presented at, or originated from, the 2015 and 2016 editions of the international meeting on *Computational Intelligence methods for Bioinformatics and Biostatistics*, CIBB2015 and CIBB2016, respectively.

The CIBB conference series provides a multi-disciplinary forum open to researchers interested in the application of computational intelligence, in a broad sense, to open problems in bioinformatics, biostatistics, systems and synthetic biology, medical informatics, as well as computational approaches to life sciences in general. In line with the spirit of CIBB, the 2015 and 2016 meetings brought together researchers from different communities who address problems from different, but connected and often overlapping, perspectives. CIBB provides them a venue to discuss cutting-edge methodologies and accelerate life science discoveries. More than 80 researchers from Europe, Asia, USA and Africa attended each the two editions, with many researchers attending both of them. Six keynote speakers, prominent scholars in their fields, were invited to present the latest advances of their research in each edition, providing insights into open problems and future directions of general interest for the field. Overall, more than 110 papers were submitted to the two editions, about 50 at CIBB2015 and 60 at CIBB2016, a positive trend confirming the interest and maturity of the CIBB series. Peer review was comprehensive and thorough thanks to the support of the Program Committee and several tens of external referees. For each edition, a large selection of presented papers was published in a post proceeding book by Springer [1, 2].

The eleven papers presented in this BMC issue were selected amongst those which received best scores in the peer review process of the conference and which were found representative of the wide range of expertise present in the two CIBB editions. In a few cases, the selected papers present results that span the two editions. Some are the result of new collaborations originated by interaction and exchanges of ideas at the meetings, sort of a collective contribution by the CIBB community. All papers underwent a further round of reviews according to the BMC standards. All reflect feedback and discussions from the meeting and extend the original results presented at the conference. Therefore, we believe that they represent a contribution not only by disseminating novel results, methods and algorithms within the community, but also by promoting the CIBB conference within the larger bioinformatics and biostatistics communities.

## CIBB 2015 and 2016 editions

CIBB2015 [1] was held in Naples, IT, from the 10 to the 12 September 2015 (http://bioinfo.na.iac.cnr.it/cibb2015/). This edition was particularly focused in different areas of bioinformatics and biostatistics to discuss recent advances, future perspectives and challenges aimed to have a high impact on molecular biology and translational medicine. Participants in CIBB2015 came from statistics, bioinformatics and bio-medical backgrounds and institutions, both from academia and the private sector. In particular, CIBB2015 offered collaboration opportunities and discussed novel results in areas of computational life sciences, and applied biologists were invited to join the discussion to propose novel challenges aimed to have a high impact for molecular biology and translational medicine. CIBB2015 also hosted five special sessions on specific themes: enhanced definition of genomic entities for systems biomedicine in oncology, metabolic models and statistical bioinformatics of adaptations and biological association, large-scale and HPC data analysis in bioinformatics, power of data analysis and integration method, regularization methods for genomic data analysis.

CIBB2016 [2] was held in Stirling, UK, from the 1st to the 3rd September 2016 (http://www.cs.stir.ac.uk/events/cibb2016/). This meeting particularly addressed advances and future perspectives in different areas, and fostered interaction between theory and practice. Results about methodologies used to model and analyse biological systems, about the practical applications of such models and approaches, as well as supporting technologies were discussed at the meeting. Participants in CIBB2016 came from mathematical, computational and medical backgrounds and institutions, both from academia and the private sector. CIBB2016 offered collaboration opportunities and novel results in areas of computational life sciences. Emerging and strongly developing trends and future opportunities at the edge of mathematics, computer and life sciences were discussed at the meeting, including synthetic biology, statistical investigation of genomic data, and applications to the understanding of complex diseases, such as cancer, and therapy opportunities. CIBB2016 also hosted six special sessions on specific themes: biomedical databases, synthetic cell biology, high-performance computing in genetics, modelling for systems biology and medicine, survival analysis and statistical inference in biological models.

## CIBB history

From 2004 to 2007, CIBB has had the format of a special session of larger conferences, namely, WIRN 2004 in Perugia, WILF 2005 in Crema, FLINS 2006 in Genoa, and WILF 2007 in Camogli. Given the great success of the special session at WILF 2007 that included 26 strongly rated papers, the Steering Committee decided to turn CIBB into an autonomous conference starting with the 2008 edition in Vietri. The following editions in Italian venues were held in Genoa (2009), Palermo (2010) and Gargnano (2011). Until 2012, CIBB meetings were held annually in Italy with an increasing number of participants. CIBB 2012 was the first edition organized outside Italy, in Houston, then in Nice, France (2013), Cambridge, UK (2014), Naples, Italy (2015) and Stirling, UK (2016). By the continued support of the community, the 2017 edition of CIBB was held in Cagliari, Italy in the meanwhile this issue was organized, and the 2018 conference will be organized in Lisbon, Portugal.

The CIBB conference series is made possible by the efforts of the organising, program and steering committees and by the support of sponsors and participants. A rigorous peer-review selection process is applied every time to ultimately select the papers included in the program of the conference, in the post-conference Proceedings published by LNBI-LNCS book series by Springer-Verlag, and in some cases, selected papers were published in special issues of well-qualified international journals, such as BMC Bioinformatics.
